# The clinical features of anti-CNTN1 antibodies associated autoimmune nodopathies with nephropathy: a case series

**DOI:** 10.3389/fneur.2026.1801275

**Published:** 2026-09-03

**Authors:** Jinping Wan, Tiegen Xiong, Jianian Hu, Shaojun Liu, Jie Lin, Chongbo Zhao, Chong Sun, Hai Xiao

**Affiliations:** 1 Department of Neurology, Guigang City People’s Hospital, Guigang, Guangxi, China; 2 Department of Neurology, General Hospital of Southern Theater Command, Guangzhou, Guangdong, China; 3 Department of Neurology, Huashan Hospital, Fudan University, Shanghai, China; 4 Rare Disease Center, Huashan Hospital, Fudan University, Shanghai, China; 5 National Center for Neurological Disorders (NCND), Shanghai, China; 6 Department of Nephrology, Huashan Hospital, Fudan University, Shanghai, China

**Keywords:** autoimmune nodopathy, contactin-1, lupus nephritis, membranous nephropathy, nephropathy

## Abstract

**Objective:**

The concurrence of anti-contactin-1 (CNTN1) antibody-associated autoimmune nodopathies (AN) and nephropathy has been sporadically reported, though the underlying relationship remains unclear. This case series study aimed to investigate the clinical characteristics and renal pathology of anti-CNTN1 antibody-associated AN with nephropathy.

**Methods:**

A retrospective case series was conducted including 69 patients diagnosed with AN, among whom 11 patients tested positive for anti-CNTN1 antibodies. Clinical manifestations, electrophysiological data, laboratory results, and renal biopsy findings were collected and analyzed. The presence of nodal/paranodal antibodies was confirmed using serum cell-based assays. Renal biopsies were performed in four patients, with immunohistochemical and immunofluorescence staining, including CNTN1 staining, to characterize renal pathology.

**Results:**

Compared to patients with non-anti-CNTN1 antibody-associated AN, the anti-CNTN1 antibody group had a significantly lower proportion of males (54.5% vs. 84.5%, *p* = 0.024) but showed higher rates of hypertension (36.4% vs. 8.6%, *p* = 0.021) and positive autoimmune antibodies (50% vs. 5%, *p* < 0.001). This group also exhibited a markedly higher prevalence of proteinuria (63.6% vs. 10.3%, *p* < 0.001) and nephropathy (63.6% vs. 3.4%, *p* < 0.001). Among anti-CNTN1 antibody-positive patients, females were more prone to concomitant nephropathy. Patients with both conditions exhibited onset ages ranging from 17 to 67 years, with a chronic course, frequent positive Romberg’s sign, and prolonged distal motor and F-wave latencies. Renal biopsy in four patients revealed membranous nephropathy (MN) in three cases and lupus nephritis (LN) in one case. Notably, CNTN1 staining of kidney tissue was positive only in patients with anti-CNTN1 antibody-associated AN with MN.

**Conclusion:**

Anti-CNTN1 antibody-associated AN patients have a higher prevalence of nephropathy compared to those AN without anti-CNTN1 antibodies. Females demonstrate a particularly higher risk of comorbid AN and nephropathy. Renal pathology in these patients is not limited to MN but can also include LN. These findings highlight a complex immunopathological relationship between anti-CNTN1 antibody-associated AN and nephropathy, warranting further studies to understand the underlying mechanisms.

## 1 Introduction

Autoimmune nodopathies (AN) represent a category of immune-mediated neuropathies associated with antibodies directed against cell adhesion molecules at the node of Ranvier and the paranodal structure (1, 2). Antibodies associated with AN include anti-contactin 1 (CNTN1) antibody (3), anti-neurofascin 155 (NF155) antibody (4), anti-contactin-associated protein 1 (Caspr1) antibody (5), and anti-pan-neurofascin (NF186/140) (6). Given that patients with AN exhibit specific clinical, pathological, and treatment response profiles, they have been classified into a distinct diagnostic category in the European Academy of Neurology/Peripheral Nerve Society chronic inflammatory demyelinating polyradiculoneuropathy (CIDP) diagnostic guidelines (2).

CNTN1 is an axonal adhesion molecule that interacts with Caspr1 on the axon and NF155 on the myelin sheath (3, 5). In 2013, researchers first confirmed the presence of anti-CNTN1 IgG4 antibodies in CIDP patients (3). Anti-CNTN1 antibody-associated AN is a significant subtype within the AN spectrum that exhibits distinct specificity in its clinical phenotype: acute or subacute onset with rapid progression, sensory-motor neuropathy characterized by predominant distal weakness and sensory ataxia; cranial nerve involvement and neuralgia may also occur, and there is often a poor response to intravenous immunoglobulins (IVIG) (7). Notably, a key clinical feature that distinguishes this subtype from other antibody-associated AN subtypes is its high tendency for comorbidity with nephropathy. Studies have shown that 45.5 to 80% of patients with anti-CNTN1 antibody-associated AN develop concurrent nephrotic syndrome (NS) (8–11), whereas this proportion is significantly lower in other AN subtypes. This comorbid relationship exhibits a complex temporal pattern: nephropathy may occur prior to, synchronously with, or subsequently after AN (3, 8–13). The temporal diversity observed suggests that there may be a shared immunological pathogenesis between the two conditions, rather than a simple causal relationship.

Despite the ongoing deepening of research regarding the association between anti-CNTN1 antibody-associated AN and nephropathy, notable limitations persist within this research field. To date, only case reports and a limited number of case series involving patients with this condition have been published. In this study, we retrospectively analyzed the comorbidity of nephropathy in 11 patients with anti-CNTN1 antibody-associated AN, aiming to summarize the clinical characteristics of nephropathy in this patient population.

## 2 Methods

### 2.1 Patients

We retrospectively included 69 patients with anti-nodal/paranodal antibodies identified through routine clinical testing of nodal/paranodal antibodies for which clinical information was available. The diagnosis of AN for the enrolled participants was clinically and serologically confirmed, as detailed in our previous study (14). These patients were admitted to the Department of Neurology and the Department of Nephrology at Huashan Hospital, affiliated to Fudan University in Shanghai, between January 2018 and December 2024. Among all AN patients, 11 patients had anti-CNTN1 antibody-associated AN, and 9 of these patients had nephropathy. All patients diagnosed with nephropathy were confirmed through consultations with nephrology experts. All patients exhibited massive proteinuria and hypoproteinemia, which met the diagnostic criteria for NS. This study received approval from the Ethics Committee at Huashan Hospital, Fudan University. All patients provided written informed consent to participate in the study.

### 2.2 Clinical data collection

Clinical data of patients with AN were collected by trained neurologists. Demographic data included gender, age of onset, triggering infection or vaccination, history of diabetes, and other autoimmune diseases. Clinical features, such as onset type, initial and clinical symptoms, symmetrical onset, complications, and clinical signs, were collected. Information about ancillary tests, including serum albumin level, proteinuria, and 24-h urinary protein quantity, was also collected. The results of cerebrospinal fluid (CSF) examination and routine nerve conduction studies (NCS) were also recorded.

### 2.3 Pathological examinations for renal biopsy specimens

**Figure 1:**
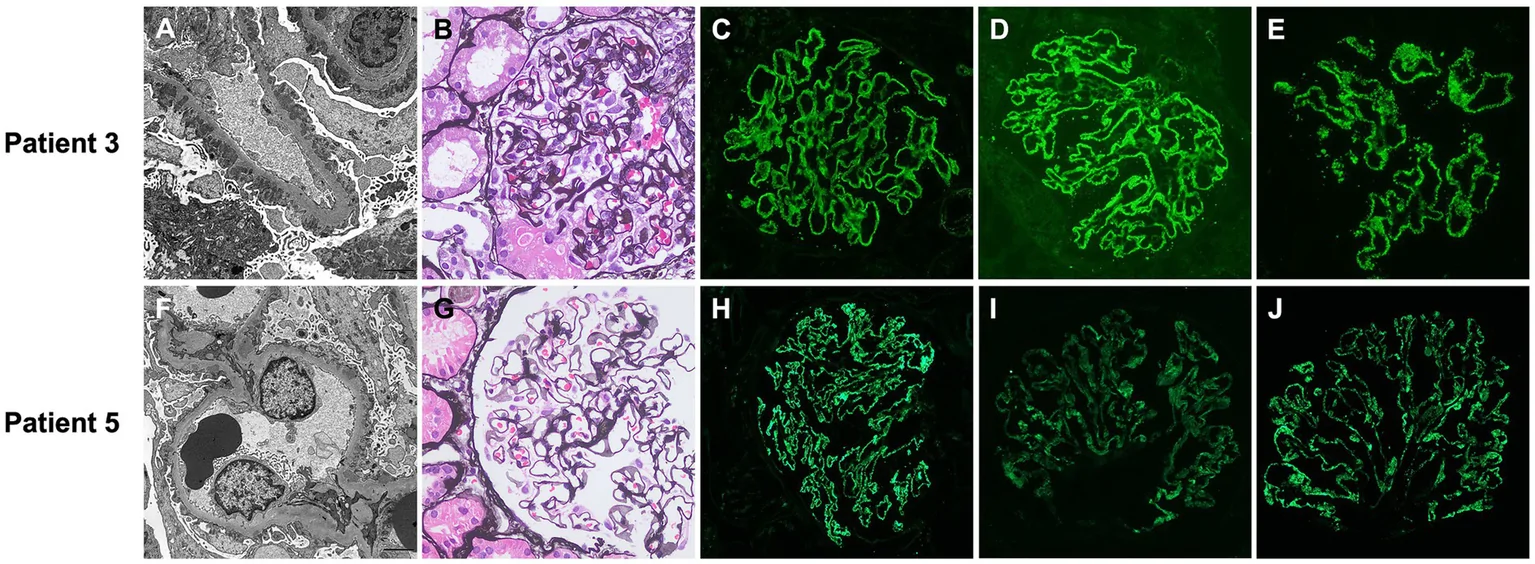
Renal pathology findings in patient 3 and patient 5. **(A,F)** Electron microscopy showed diffuse thickening of the glomerular basement membrane, and abundant electron-dense deposits beneath the epithelium and within the basement membrane, with a portion of the electron-dense matter embedded and absorbed in the basement membrane. **(B,G)** Periodic acid-methenamine silver (PAM, x400) staining of the patients^,^ glomeruli revealed a spiked appearance of the glomerular basement membrane. Immunofluorescence microscopy (x400) demonstrates granular deposits of IgG **(C,H)**, IgG1 **(D,I)** and IgG4 **(E,J)** along the glomerular basement membrane.

Renal biopsies were obtained from four patients with anti-CNTN1 antibody-associated AN and nephropathy. Pathological data of anti-CNTN1 antibody seronegative patients with nephropathy (one with lupus nephritis and one with membranous nephropathy) were included as controls. Ultimately, kidney tissue sections for CNTN1 staining were available from only two patients. Paraffin tissue was stained with hematoxylin and eosin (HE), periodic acid-silver methenamine (PASM), periodic acid-Schiff (PAS), and Masson trichrome. Immunofluorescence was performed on cryostat sections using a panel of FITC-conjugated rabbit anti-human antibodies to IgG, IgM, IgA, C3, and C1q. We performed immunofluorescence staining for CNTN1 on renal paraffin sections from two patients with anti-CNTN1 antibody-associated AN combined with NS and two patients with nephropathy not associated with anti-CNTN1 antibodies. The specific steps of immunofluorescence staining for CNTN1 in renal pathological tissues are as follows. Firstly, tissue sections were deparaffinized, rehydrated, underwent antigen retrieval by microwave, and then blocked with phosphate-buffered saline (PBS). Secondly, sections were incubated overnight at 4 °C with goat anti-CNTN1 antibody (AF904, R&D Systems, 1:300), followed by incubation with biotinylated anti-goat FITC antibody (product number 305–095-003, Jackson ImmunoResearch Laboratories, 1:100) at room temperature for 1 h. Lastly, the sections were washed three times with PBS on a shaker for 5 min each and then mounted with glycerol (Figure 1).

### 2.4 Statistical analysis

Descriptive statistics are shown as mean (±SD) for continuous variables and as frequencies (percentage) for categorical variables. Statistical analysis was conducted with SPSS 27.0 statistical software. A *p*-value < 0.05 was defined as statistically significant.

## 3 Results

### 3.1 Clinical features of the anti-CNTN1 antibody associated AN patients

We divided all AN patients into an anti-CNTN1 antibody-associated AN group and a non-anti-CNTN1 antibody-associated AN group, then compared the general clinical characteristics and comorbidities of nephropathy between the two groups. As shown in Table 1, there were no statistically significant differences between the anti-CNTN1 antibody-associated AN group and the non-anti-CNTN1 antibody-associated AN group in terms of age at onset and the prevalence of diabetes and other autoimmune diseases. Meanwhile, there were no statistically significant differences between the two groups regarding triggering infection or vaccination, onset form, onset site, initial symptom, cranial nerve involvement, and CSF protein content. However, the proportion of males in the anti-CNTN1antibody-associated AN group was lower than that in the non-anti-CNTN1 antibody-associated AN group (54.5% vs. 84.5%, *p* = 0.024), while the prevalence of hypertension and positive autoimmune antibodies were higher (36.4% vs. 8.6%, *p* = 0.021) and (50% vs. 5%, *p* < 0.001), respectively. Similarly, the incidence of proteinuria in the anti-CNTN1 antibody-associated AN group was higher than that in the non-anti-CNTN1 antibody-associated AN group (63.6% vs. 10.3%, *p* < 0.001), and the incidence of nephropathy was also higher (63.6% vs. 3.4%, *p* < 0.001).

**Table 1:** Comparisons of anti-CNTN1 antibody associated AN patients and non-anti-CNTN1 antibody associated AN patients.

Clinical data	Anti-CNTN1 antibody associated AN (*N* = 11)	non-anti-CNTN1 antibody associated AN (*N* = 58)	*p*-value
Gender (male)	6 (54.5%)	49 (84.5%)	0.024
Onset age (year)	51.7 ± 14.5	33.1 ± 18.3	0.166
Hypertension	4 (36.4%)	5 (8.6%)	0.021
Diabetes	2 (18.2%)	3 (5.2%)	0.127
Malignant tumor	1 (9.1%)	1 (1.7%)	0.182
Other autoimmune diseases	1 (9.1%)	1 (1.7%)	0.182
Triggering infection/vaccination	1 (9.1%)	12 (20.7%)	0.367
Onset form (acute/subcute onset)	1 (9.1%)	11 (17.5%)	0.115
Site of onset			0.887
Upper limbs	1 (9.1%)	8 (13.8%)	
Lower limbs	9 (81.8%)	44 (75.9%)	
Four limbs	1 (9.1%)	4 (6.9%)	
Cranial nerve	0 (0%)	2 (3.4%)	
Symmetrical onset	8 (80%)	49 (86.0%)	0.625
Onset symptom			0.130
Numbness	6 (54.5%)	26 (44.6%)	
Weakness	1 (9.1%)	18 (31.0%)	
Numbness and weakness	4 (36.4%)	8 (13.8%)	
Other	0 (0%)	6 (10.3%)	
Cranial nerve involvement	3 (27.3%)	11 (19.0%)	0.530
Nephropathy	7 (63.6%)	2 (3.4%)	<0.001
Proteinuria	7 (63.6%)	6 (10.3%)	<0.001
Serum creatinine level	57.0 ± 12.9	61.4 ± 16.8	0.774
Positive autoimmune antibodies	5/10 (50%)	2/40 (5%)	<0.001
CSF protein level (mg/l)	4590.1 ± 4024.9	3375.3 ± 3166.7	0.537

CNTN1, contactin-1; CSF, cerebrospinal fluid.

### 3.2 Clinical characteristics of anti-CNTN1 antibody associated AN with nephropathy patients

A total of 11 patients with anti-CNTN1 antibody-associated AN were included, among whom 7 patients had concurrent nephropathy (7/11). Female patients were more common in those with nephropathy than in those without nephropathy (5/7 vs. 1/4), and the incidence of proteinuria was higher (7/7 vs. 0/4) in patients with nephropathy. Due to the small sample size in both groups, only descriptive analyses were performed; statistical comparisons were not conducted.

**Figure 2:**
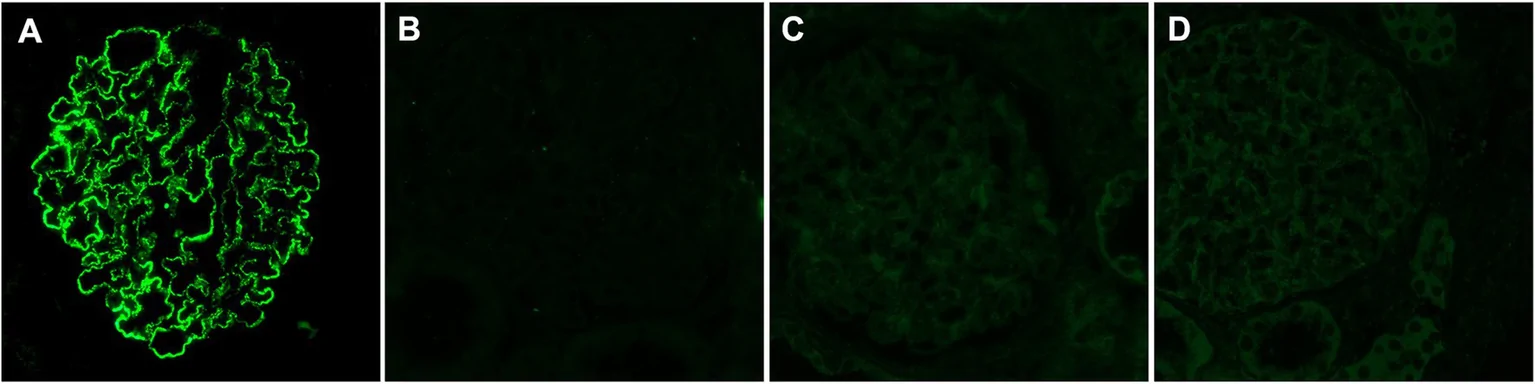
Immunofluorescence findings in the renal biopsy of patient 3 and patient 5. **(A)** Immunofluorescence microscopy (x400) demonstrates deposits of CNTN1 along the glomerular basement membrane in patient 5. The immunofluorescence of CNTN1 was negative in patient 3 **(B)**, control 1 **(C)**, who was diagnosed as MN associated with PLA2R, and control 2 **(D)**, who was diagnosed as lupus nephritis.

The clinical characteristics of seven patients with anti-CNTN1 antibody-associated AN complicated by nephropathy are presented in Table 2. Most patients with anti-CNTN1 antibody-associated AN complicated by nephropathy were middle-aged or elderly (6/7), but one child also had this comorbidity. In addition, women were more common among patients with anti-CNTN1 antibody-associated AN and nephropathy (5/7). Six patients had symmetric onset; only one patient had an acute/subacute onset; three patients presented with tremor. All six patients with recorded Romberg’s sign on physical examination tested positive, while one patient had no record of this sign. The anti-CNTN1 antibody titers in these seven patients ranged from 1:10 to 1:1000. All six patients with detailed electromyography records exhibited prolonged distal motor nerve latency and prolonged latency or absence of F-waves. Furthermore, all seven patients who underwent lumbar puncture exhibited CSF albuminocytologic dissociation. Among the seven patients, the temporal sequence of AN and nephropathy was as follows: two cases had nephropathy preceding AN; four cases had simultaneous onset; one case initially presented with AN and was diagnosed with NS 1.5 years later. Two patients with anti-CNTN1 antibody-associated AN and NS underwent serum phospholipase A2 receptor (PLA2R) antibody testing; all results were negative. Among the seven patients, only four underwent renal biopsy. Pathological results revealed membranous nephropathy (MN) in three patients and lupus nephritis (LN) in one patient. The patient with LN was a boy presenting with multiple positive autoimmune antibodies (Figure 2).

**Table 2:** Characteristics of patients with anti-CNTN1 antibody associated AN and nephrotic syndrome.

Patient ID	P1	P2	P3	P4	P5	P6	P7
Gender	Male	Female	Male	Female	Female	Female	Female
Age (years)	53	48	17	48	63	67	59
Temporal relationship between NS and AN	AN after NS	Concurrence	Concurrence	AN after NS (4 years)	NS after AN (1.5 years)	Concurrence	Concurrence
Onset form	Chronic	Chronic	Acute/subacute	Chronic	Chronic	Chronic	Chronic
Tremor	Yes	Yes	No	No	No	No	Yes
Romberg’s sign	Yes	Yes	ND	Yes	Yes	Yes	Yes
CSF protein level (mg/l) /CSF cell count*10^6/L	2420/20	2580/1	1113/10	3327/2	3097/7	5525/5	2425/5
Serum anti-CNTN1 antibody titer	1:100	1:320	1:32	1:320	1:1000	1:320	1:10
Prolonged DML	Yes	Yes	ND	Yes	Yes	Yes	Yes
Prolonged F-wave	Yes	Yes	ND	Yes	Yes	Not evoked	Not evoked
Renal pathology	MN	ND	LN	MN	ND	MN	ND
24 h-UTP (g/24 h)	ND	8.71	4.81	ND	3.5	2.36	2.52
Serum anti-PLA2R antibody	ND	Negative	Negative	ND	ND	ND	ND
Autoimmune antibodies	ND	Anti-ANA (AC-2 1:100)	Anti-ANA (AC-1 1:320, Cytoplasmic speckled pattern 1:1000), anti-dsDNA, anti-SS-A/Ro52, anti-SS-A/60, anti-SS-B/L, anti-Ku	Anti-ANA (pattern and titer ND)	Negative	Anti-ANA (Cytoplasmic speckled pattern 1:100)	Negative

AN, autoimmune nodopathy; ANA, antinuclear antibody; CNTN1, contactin-1; DML, distal motor latency; LN, lupus nephritis; MN, membranous nephropathy; ND, not determined; NS, nephrotic syndrome; P, patient; PLA2R, phospholipase A2 receptor; UTP, urine total protein.

### 3.3 Results of renal biopsy and immunofluorescence testing

The pathological characteristics of the kidneys are detailed in Table 3. A total of four patients underwent renal biopsy; three patients (Patient 1, Patient 2, and Patient 5) were diagnosed with MN, and one patient was diagnosed with LN. Complete renal pathology and immunofluorescence data for CNTN1 were available for only two patients. Immunofluorescence demonstrated variable degrees of glomerular deposition of IgG, IgM, IgA, C3, C1q, kappa chain, lambda chain, IgG1, IgG4, and PLA2R (Table 3). Patient 3 exhibited a classical full-house immunofluorescence pattern (positivity for IgG, IgG1, IgG4, C3, and C1q) along the glomerular basement membrane (GBM) and within the mesangium. Immunofluorescence staining for CNTN1 was performed on deparaffinized renal tissue sections from Patient 3 and Patient 5, as well as two anti-CNTN1 antibody seronegative patient controls (one with LN and one with MN). CNTN1 immunoreactivity was detected only in the glomeruli of Patient 5, who was an anti-CNTN1 antibody seropositive patient, and was absent in both LN and MN patients without anti-CNTN1 antibodies.

**Table 3:** Pathological features of renal biopsy in 2 patients.

The items of pathological staining	IgA	IgM	IgG	IgG1	IgG4	C1q	C3	Kappa	Lambda	PLA2R	CNTN1
Patient 3	−	−	+++	+++	+++	+	++	+++	+++	−	−
Patient5	±	−	+++	+++	+++	−	−	+++	+++	−	+++

-, negative; ±, borderline; +, weakly positive; ++, moderately positive; +++, strongly positive.

CNTN1, contactin-1; Ig, immunoglobulin; PLA2R, phospholipase A2 receptor.

Kappa, Kappa light chain; Lamda, Lamda light chain.

## 4 Discussion

We found that the proportion of males among patients with anti-CNTN1 antibody-associated AN was lower than that among patients with non-anti-CNTN1 antibody-associated AN, which appears inconsistent with previous studies. Previous literature reports indicate that anti-CNTN1 antibody-associated AN is more common in middle-aged and elderly males (3–8, 10, 15). This discrepancy may actually reflect the complex heterogeneity of this disease. Firstly, anti-CNTN1 antibody-associated AN is a rare disease subtype, with a detection rate of only 1.5–4.3% in large CIDP cohorts (3, 16). Another reason for this result is that the non-anti-CNTN1 antibody-associated AN patients in our study were those with anti-NF-155 antibody-associated AN, a subtype commonly found in young males (15).

As an early and sensitive indicator of structural or functional impairment of the glomerular filtration barrier, proteinuria is a key symptom of NS. Proteinuria has been reported as a possible biomarker of AN, especially associated with autoantibodies targeting CNTN1 (17). In this study, the incidence of proteinuria in the anti-CNTN1 antibody-associated AN group was higher than in the non-anti-CNTN1 antibody-associated AN group, consistent with the occurrence trend of NS.

Previous studies have reported that patients with non-anti-CNTN1 antibody-associated AN, especially those with anti-NF186 antibody-associated AN, can also be complicated with NS (5, 18). Our study reveals that the incidence of nephropathy in anti-CNTN1 antibody-associated AN patients is significantly higher than that in non-anti-CNTN1 antibody-associated AN patients. The difference between this study and previous studies is that the previous research subjects were mainly CIDP patients, whereas our study focused on AN patients with definite nodal or nodal-paranodal antibodies. In our study, we found two patients with anti-NF186 antibody-associated AN complicated with NS, and in one of them, renal pathology showed focal segmental glomerulosclerosis (FSGS). This finding has been previously published and is consistent with the results of earlier studies (18).

As early as 1994, a study detected the mRNA of contactin in human kidney tissues (18), while in 2021, Professor Quintrec et al. further confirmed the presence of CNTN-1 protein in normal human kidney tissues and indicated that CNTN1 protein is expressed by podocytes in the kidney (10). Subsequently, researchers conducted an in-depth analysis of renal biopsy pathological tissues from six patients with anti-CNTN1 positive AN complicated by NS, finding immune complexes containing CNTN-1 distributed along the glomerular basement membrane. Proteomics analysis further confirmed the presence of polypeptide sequences consistent with recombinant CNTN1 protein in renal tissues (8). These findings provide strong evidence illustrating the potential molecular mechanism underlying the significantly increased incidence of nephropathy in AN patients positive for anti-CNTN1 antibody.

Previous studies have confirmed that the anti-CNTN1 antibody is commonly present in patients with anti-CNTN1 antibody-associated AN and MN (8–14), but when and how the anti-CNTN1 antibody causes the coexistence of AN and MN remain unknown. Since both AN and NS mediated by anti-CNTN1 antibody are rare diseases with few cases, large-scale clinical studies are difficult to perform. In our case series, the proportion of anti-CNTN1 antibody-associated AN complicated with nephropathy was 63.6%, slightly lower than the 80% reported by Fehmi et al. (8). One study summarized 12 previously reported cases of anti-CNTN1 antibody-associated AN complicated with NS (or MN) and found that only five cases (41.6%) had detectable autoimmune antibodies, including lupus anticoagulant antibody, anti-PLA2R antibody, anti-SS-A/Ro antibody, and anti-ANA antibody (11). In our study, the detection rate of autoimmune antibodies in anti-CNTN1 antibody-associated AN patients was 40%. Moreover, we found that among these patients, the coexistence of autoimmune antibodies was more common in those with NS. This may suggest that anti-CNTN1 antibody-associated AN is likely complicated by other autoimmune diseases, and additional autoimmune antibodies may contribute to anti-CNTN1-related kidney damage. This hypothesis requires further research to confirm.

In our study, the proportion of female patients with anti-CNTN1 antibody-associated AN complicated with NS was higher (5/7), which contrasts with previous studies (8–14). This discrepancy may be due to publication bias and a limited sample size.

To date, only one boy with anti-CNTN1 antibody-associated AN and NS has been reported (19), with most previous case reports involving middle-aged and elderly patients with MN confirmed by renal biopsy (8–13, 20, 21). In our case series, we identified an adolescent male whose renal biopsy revealed LN. This patient was seropositive for multiple autoimmune antibodies, including anti-ANA, anti-dsDNA, anti-SS-A/Ro52, SS-A/Ro60, anti-SS-B/La, and anti-Ku antibodies, but seronegative for anti-PLA2R antibody. The clinical manifestation of renal damage was NS, and renal biopsy pathology classified LN as type V according to the ISN/RPS classification. Based on the diagnostic criteria of the European League Against Rheumatism (EULAR) and the American College of Rheumatology (ACR) (22), this case was diagnosed as SLE complicated with AN. A similar case was reported by Shida et al. (11) involving a patient with anti-CNTN1 antibody-associated AN and nephropathy complicated by multiple positive autoimmune antibodies simultaneously. However, the renal biopsy pathology of that patient mainly showed MN, with CNTN-1 staining distributed along the glomerular basement membrane. Immunohistochemical and immunofluorescence staining showed a full-house pattern, suggesting possible concurrent LN. Unlike that case, immunofluorescence of renal pathology in our patient showed a full-house pattern along the glomerular basement membrane (GBM) and mesangial regions, but CNTN1 staining was negative, leading to a final diagnosis of LN. This suggests that nephropathy associated with anti-CNTN1 antibody is not limited to MN but may also include LN, especially when anti-CNTN1 antibody along with anti-dsDNA antibody were detected in the serum. For patients with SLE, their specific anti-dsDNA antibodies can deposit in the glomeruli, basement membranes and mesangial areas of the kidneys, thereby causing kidney damage (23).

Autoimmune antibodies reactive with neural antigens were detected in 13.2% (24/174) of SLE patients, and among these seropositive individuals, 56.5% (13/24) manifested clinical features consistent with CIDP (22). Although definite evidence linking anti-CNTN1 antibody production directly to SLE disease activity is lacking, we believe that SLE and AN can coexist in rare cases.

Our study has several limitations. First, as a retrospective study, the clinical data of some patients are incomplete, including specific immunohistochemical and immunofluorescence staining results of renal biopsy and the determination of anti-PLA2R antibody. Second, not all patients with nephropathy underwent renal biopsy to confirm the pathological type. Third, the number of patients with anti-CNTN1 antibody-associated AN is too small to perform statistical analysis.

In conclusion, our study shows that the prevalence of nephropathy in patients with anti-CNTN1 antibody-associated AN is much higher than in those with non-anti-CNTN1 antibody-associated AN. Additionally, we found that females are more common among patients with anti-CNTN1 antibody-associated AN and nephropathy. Regardless of the chronological order of nephropathy and AN, proteinuria always occurs in these patients. This suggests that proteinuria may serve as a sensitive indicator for monitoring the development of nephropathy in patients with anti-CNTN1 antibody-associated AN. We also observed that anti-CNTN1 antibody-associated AN concurrent with nephropathy can occur in children. Finally, in patients with anti-CNTN1 antibody-associated AN and nephropathy, renal pathology includes not only MN but also LN, with partial CNTN1 staining observed in renal biopsies. This suggests that the relationship between anti-CNTN1 antibody and nephropathy is not merely antibody-mediated kidney injury, especially when anti-CNTN1 antibody coexists with multiple autoimmune antibodies. Further studies with larger sample sizes are needed to clarify the relationship and pathogenesis between anti-CNTN1 antibody-associated AN and nephropathy.

## Data Availability

The raw data supporting the conclusions of this article will be made available by the authors, without undue reservation.
